# Intestinal bitter taste receptor activation alters hormone secretion and imparts metabolic benefits

**DOI:** 10.1016/j.molmet.2018.07.013

**Published:** 2018-08-04

**Authors:** Bernard P. Kok, Andrea Galmozzi, Nicole K. Littlejohn, Verena Albert, Cristina Godio, Woojoo Kim, Sean M. Kim, Jeffrey S. Bland, Neile Grayson, Mingliang Fang, Wolfgang Meyerhof, Gary Siuzdak, Supriya Srinivasan, Maik Behrens, Enrique Saez

**Affiliations:** 1 Department of Molecular Medicine, USA; 2 Center for Metabolomics, The Scripps Research Institute, 10550 North Torrey Pines Road, La Jolla, CA, 92037, USA; 3 Kindex Pharmaceuticals, 800 Fifth Avenue, Seattle, WA, 98104, USA; 4 Department of Molecular Genetics, German Institute of Human Nutrition Potsdam-Rehbruecke, Arthur-Scheunert-Allee 114-116, 14558, Nuthetal, Germany; 5 Leibniz-Institute for Food Systems Biology at the Technical University of Munich, Lise-Meitner-Str. 34, 85354, Freising, Germany

**Keywords:** Bitter taste receptor, Diabetes, Enteroendocrine hormones, GLP-1, Intestinal signaling, Isohumulone

## Abstract

**Objectives:**

Extracts of the hops plant have been shown to reduce weight and insulin resistance in rodents and humans, but elucidation of the mechanisms responsible for these benefits has been hindered by the use of heterogeneous hops-derived mixtures. Because hop extracts are used as flavoring agents for their bitter properties, we hypothesized that bitter taste receptors (Tas2rs) could be mediating their beneficial effects in metabolic disease. Studies have shown that exposure of cultured enteroendocrine cells to bitter tastants can stimulate release of hormones, including glucagon-like peptide 1 (GLP-1). These findings have led to the suggestion that activation of Tas2rs may be of benefit in diabetes, but this tenet has not been tested. Here, we have assessed the ability of a pure derivative of a hops isohumulone with anti-diabetic properties, KDT501, to signal through Tas2rs. We have further used this compound as a tool to systematically assess the impact of bitter taste receptor activation in obesity-diabetes.

**Methods:**

KDT501 was tested in a panel of bitter taste receptor signaling assays. Diet-induced obese mice (DIO) were dosed orally with KDT501 and acute effects on glucose homeostasis determined. A wide range of metabolic parameters were evaluated in DIO mice chronically treated with KDT501 to establish the full impact of activating gut bitter taste signaling.

**Results:**

We show that KDT501 signals through Tas2r108, one of 35 mouse Tas2rs. In DIO mice, acute treatment stimulated GLP-1 secretion and enhanced glucose tolerance. Chronic treatment caused weight and fat mass loss, increased energy expenditure, enhanced glucose tolerance and insulin sensitivity, normalized plasma lipids, and induced broad suppression of inflammatory markers. Chronic KDT501 treatment altered enteroendocrine hormone levels and bile acid homeostasis and stimulated sustained GLP-1 release. Combined treatment with a dipeptidyl peptidase IV inhibitor amplified the incretin-based benefits of this pure isohumulone.

**Conclusions:**

Activation of Tas2r108 in the gut results in a remodeling of enteroendocrine hormone release and bile acid metabolism that ameliorates multiple features of metabolic syndrome. Targeting extraoral bitter taste receptors may be useful in metabolic disease.

## Highlights

• A pure analog of hops isohumulones signals via the bitter taste receptor Tas2r108.
• KDT501 stimulates endogenous GLP-1 secretion and enhances glucose tolerance.
• Long-term KDT501 treatment reduces weight, dyslipidemia, and insulin resistance.
• KDT501 action *in vivo* is augmented by sitagliptin, a DPP-IV inhibitor.

## 1 Introduction

Extracts of the hops plant (*Humulus lupulus* L.*)* used as flavoring agents in beer production have been shown to reduce weight and improve glucose homeostasis in rodents [1], [2], [3], [4], [5], [6], [7], [8] and humans [1], [9], [10]. Thus, it is of considerable interest to identify the molecular mechanisms that mediate the benefits of bioactive ingredients of hops in metabolic disease. The humulones or α-acids are among the most abundant phytochemicals synthesized by female hop cones [11]. Hop α-acids are isomerized during brewing to iso-α-acids, or isohumulones, that impart beer its characteristic bitter flavor. There are three main isohumulones (*n*-humulone, cohumulone, and adhumulone) that account for more than 80% of the hops-derived matter in beer [11]. Each isohumulone occurs as a mixture of *cis* and *trans* isomers that can be reduced into rho, tetrahydro, or hexahydro analogs. This level of chemical complexity has hampered efforts to uncover the mechanism of action of isohumulones in metabolic syndrome.

An initial study [1] with mixtures of isohumulones showed that obese-diabetic KK-*A*^y^ mice treated with these compounds had reduced glycemia and decreased plasma triglycerides and free fatty acid levels. Similarly, mice with established Diet Induced Obesity (DIO) treated with isohumulones showed enhanced glucose tolerance and insulin sensitivity. These benefits were ascribed to direct activation of peroxisome proliferator-activated receptors (PPARs) α and γ. However, no changes in PPARγ target gene expression could be detected in isohumulone-treated mice, suggesting that isohumulones exert their benefits through alternative, unknown mechanisms. Interestingly, this study also included a small double-blind, placebo-controlled trial in people with diabetes that showed that treatment for 8 weeks with isohumulones significantly reduced blood glucose and hemoglobin A1c levels. Subsequent studies [2], [3], [4], [5], [8] have reported positive effects of isohumulones in reducing weight gain and fat mass, improving glucose homeostasis and insulin sensitivity, decreasing liver lipid accumulation, and reducing plasma triglycerides in multiple rodent models (e.g., DIO, KK-*A*^y^, *db/db* mice, Wistar rats). Notably, a larger clinical trial in Japanese subjects with prediabetes showed that 12-week treatment with isohumulones significantly reduced body weight, body mass index, fat mass, fasting blood glucose, and hemoglobin A1c levels [9].

An important, enabling development towards the identification of the molecular players responsible for the effects of isohumulones was the synthesis of KDT501, a stereochemically pure substituted 1,3-cyclopentadione [12]. This derivative of hops tetrahydro iso-α-acids retained the desirable properties of natural isohumulone mixtures [13]. DIO mice and Zucker Diabetic Fatty rats treated orally with KDT501 showed decreased weight and fat mass, increased insulin sensitivity, and reduced plasma lipids. This study, however, did not decisively determine the molecular target(s) of KDT501 relevant for its action.

Given that hops-derived compounds are used as flavoring agents for their bitter properties, it is conceivable that the benefits of KDT501 and natural isohumulones on metabolic disease may be due to signaling through bitter taste receptors present in extraoral tissues, particularly the gastrointestinal (GI) tract. Beyond its role in nutrient absorption, the GI tract is emerging as a powerful regulator of systemic energy balance that secretes hormones that affect varied aspects of physiology and behavior [14], [15]. Taste receptors were discovered in the tongue [16], [17], [18], but it is now well established that they are expressed in multiple tissues besides the oral cavity, including the GI tract [19], [20]. Bitter taste receptors (mouse Tas2rs; human TAS2Rs) are G_α_-gustducin/G_q_-coupled GPCRs that were initially thought to have evolved as a defense mechanism to avoid ingestion of harmful substances [21]. However, it is now clear that in addition to this protective function in the mouth, extraoral bitter taste receptors play important roles in physiology [22], [23]. For instance, exposure of cultured enteroendocrine cells to promiscuous bitter tastants (e.g., quinine) is known to increase intracellular calcium signaling [24] and stimulate release of hormones, including cholecystokinin (CCK) [25], [26], [27], Peptide YY (PYY) [28], and glucagon-like peptide 1 (GLP-1) [26], [29]. The ability of bitter compounds to acutely stimulate intestinal hormone secretion, particularly incretins, has led to the suggestion that activation of Tas2rs may be of benefit in diabetes [30]. However, the extent to which these effects may be seen *in vivo* and in sufficient magnitude to be therapeutic is not known. Unfortunately, evaluation of the therapeutic potential of bitter taste receptor ligands has been hampered by a lack of selective Tas2r agonists. Quinine, for instance, signals through Tas2rs but is also known to activate the calcium-activated cation channel Trpm5 [31], several potassium channels [32], [33], and to directly enhance ERK/S6 kinase [34] and G-protein signaling [35].

In this report, we provide evidence that KDT501 and, by inference natural isohumulones, exert their anti-diabetic effects via modulation of bitter taste receptor signaling in the gut. A principal mediator of these effects is GLP-1, which has broad effects on physiology and in reversing metabolic dysfunction. We show that KDT501 activates a single mouse (Tas2r108) or human (TAS2R1) bitter taste receptor (of 35 and 25, respectively). Enteroendocrine cells exposed to KDT501 secreted GLP-1, and this effect was blunted in cells depleted of Tas2r108. The excellent selectivity of KDT501 enabled us to use this compound as a tool to examine the impact of activation of bitter taste receptor signaling in obesity-diabetes. DIO mice treated with a single dose of KDT501 had enhanced glucose tolerance. Chronic dosing resulted in weight loss, reduced fat mass, increased energy expenditure, greater glucose tolerance and insulin sensitivity, and a normalization of plasma lipid profiles accompanied by broad suppression of circulating inflammatory markers. The basis of these benefits is a remodeling of gut hormone secretion and bile acid homeostasis that includes stimulation of GLP-1 release. These findings indicate that targeting gut bitter taste receptors may be of utility in metabolic disease. Further, co-treatment with a dipeptidyl peptidase IV (DPP-IV, the enzyme that degrades GLP-1) inhibitor considerably augmented the incretin-based benefits of this pure isohumulone, stressing the potential advantage of combined administration of an incretin secretagogue (e.g., KDT501) with DPP-IV inhibitors in clinical use. Our results illustrate an interesting paradigm by which naturally occurring compounds can harness gut signaling to alter whole body metabolism.

## 2 Materials and methods

### 2.1 Animal studies

C57BL/6N DIO and control mice were purchased from Taconic Biosciences at 17 weeks of age and maintained on a 60% and 10% kcal high fat diet (Research Diets D12492, D12450J), respectively. All experiments were conducted with male mice housed (2–4 mice per cage) in ventilated rack systems under regular housing temperatures (22–24 °C) with a 12 h light/12 h dark cycle (06:00 light on to 18:00 light off). Compounds were homogenized (Tissue-Tearor, Biospec) and bath-sonicated in 0.5% methylcellulose containing 0.2% Tween-80 (vehicle). Mice were dosed daily by oral gavage with vehicle, 150 mg/kg KDT501, or 10 mg/kg rosiglitazone. The dose of KDT501 was chosen based on prior work [13]. Weights were monitored weekly and mice were fasted for 16 h prior to blood collection. Animal experiments were approved by and conducted in accordance with the guidelines of The Scripps Research IACUC.

### 2.2 Cell culture

STC-1 cells were purchased from the American Type Culture Collection (RRID CVCL_J405). Cells were cultured in 10% FBS-DMEM and maintained in a 37 °C, 5% CO_2_ incubator. Lentiviral shRNA constructs targeting mouse Tas2r108 (TRCN0000418872 and TRCN0000072142) and the corresponding controls (SHC202 and SHC002) were purchased from Sigma–Aldrich. Experimental results were similar with both shRNAs, and results from first- (TRCN0000072142) and second-generation (TRCN0000418872) TRC vectors are shown in the supplementary and main figures, respectively.

### 2.3 Plasma lipid and hormone measurements

Blood was collected from the tail of non-sedated mice, retro-orbital plexus of isoflurane-sedated mice, or by cardiac puncture upon euthanasia. The DPP-IV inhibitor KR-62436 (Sigma–Aldrich) was added at a final concentration of 5 μM for blood collection related to GLP-1 assays. Plasma triglycerides (Sigma–Aldrich), non-esterified free fatty acids (Sigma–Aldrich), ketone bodies (Bioassay Systems), and cholesterol (Wako Chemicals) were analyzed with colorimetric assay kits. Plasma insulin, GLP-1, PYY, and ghrelin levels were analyzed using luminex-based magnetic bead immunoassays (Bio-Rad and Millipore). Plasma CCK was measured using an extraction-free EIA assay (Phoenix Pharmaceuticals), and active GLP-1 was measured using an ELISA kit (Millipore).

### 2.4 Glucose and insulin tolerance tests

For glucose tolerance tests, mice were fasted for 16 h prior to oral gavage of a glucose bolus (1 g/kg). For insulin tolerance tests, mice were fasted for 4 h prior to intraperitoneal injection of insulin (0.75 U/kg). For glucose tolerance tests using an acute single dose of KDT501 and/or sitagliptin, mice were dosed orally with 150 mg/kg KDT501 or 4 mg/kg sitagliptin (Biovision Inc.) 30 min prior to an oral gavage of 2 g/kg glucose.

### 2.5 Energy balance studies

Respiration, locomotor activity, and food intake of DIO mice were monitored using Oxymax-Comprehensive Laboratory Animal Monitoring System (CLAMS) cages (Columbus Instruments) after 21 days of KDT501 treatment. Mice were acclimated for 2 days with continued KDT501 treatment. Throughout the duration of these studies, mice were maintained on the 60% kcal high fat diet. Respiratory rate was normalized to body weight 0.75.

### 2.6 Lipid analysis

Liver samples were Folch-extracted using 10 volumes of 2:1:1 chloroform-methanol-0.9% sodium chloride. Fecal samples were dried prior to extraction using the same methodology. The organic phase was dried down and resuspended in PBS containing 5% Nonidet P-40, and triglycerides were assayed using a colorimetric kit (Sigma–Aldrich). For fecal NEFA (Sigma–Aldrich) and cholesterol (Wako Chemicals) measurements, organic phase aliquots were dried down and sonicated directly in assay buffer. For total bile acid analysis, tissue and fecal samples were extracted in 2:1 chloroform-methanol. The supernatants were dried down and resuspended in PBS containing 5% Nonidet P-40 prior to colorimetric kit assay analysis (Diazyme Laboratories).

### 2.7 Calcium flux FLIPR assays

STC-1 cells (8,000 cells per well) were infected with lentiviral shRNA vectors on poly-l-lysine coated 384-well black-walled plates (CellCoat, Grenier Bio-One). 72 h after infection, cells were loaded with Fluo-8 AM (Screen Quest Fluo-8 NW calcium assay kit, AAT Bioquest). Intracellular calcium flux upon acute compound injection was measured using the FLIPR Tetra screening system (Molecular Devices). Results were expressed as relative fluorescence units (RFU) after mean fluorescence intensities from vehicle-treated wells were subtracted from that of compound-treated wells.

### 2.8 *In vitro* GLP-1 secretion assay

STC-1 cells (30,000 cells per well) were plated on poly-l-lysine coated 96 well plates. After 2 days, cells were starved in Krebs–Ringer buffer for 1 h, followed by addition of treatments in Krebs–Ringer buffer containing 5 μM of the DPP-IV inhibitor KR-62436. Media was collected after 30 min and active GLP-1 in media was measured using a GLP-1 HTRF kit (Cisbio).

### 2.9 RNAscope hybridization and imaging

Mouse intestine and colon were briefly washed in PBS and the small intestine was divided into 3 equal sections (duodenum, jejunum, and ileum). Tissues were frozen in embedding medium (Tissue Tek O.C.T. Compound; Sakura Finetek) and stored at −80 °C. Cryostat sections (10 μm) were processed for RNAscope analysis according to the manufacturer's instructions (RNAscope Multiplex Fluorescent Reagent v2 protocol for fresh frozen tissue; Advanced Cell Diagnostics). Briefly, sections were pretreated with protease IV, and probes (Chga, 447851; Il25, 474631; Tas2r108, 539889) hybridized to detect specific RNA targets. To amplify the fluorescent signal, subsequent hybridization was conducted with RNAscope detection reagents and TSA Plus fluorophores (fluorescein, Cyanine 3, and Cyanine 5; PerkinElmer). All images were captured using a Nikon A1 confocal at 60× magnification.

### 2.10 Histology and adipocyte size measurements

Tissues were fixed in 10% neutral buffered formalin (Sigma–Aldrich), dehydrated, and embedded in paraffin. Hematoxylin- and eosin-stained sections were scanned and adipocyte sizes were determined using the Adipocytes Tools macro in ImageJ software.

### 2.11 Inflammatory cytokine measurements

Markers of inflammation in plasma were measured using the Bio-Plex Pro mouse cytokine 23-plex assay (M60009RDPD; Bio-Rad).

### 2.12 Mass spectrometry analysis of KDT501 tissue distribution

Tissues were homogenized in 10 volumes of 2:2:1 methanol:acetonitrile:water using a Bullet Blender Blue, followed by 15 min of bath sonication. After storage at −20 °C for 60 min to further precipitate the protein, samples were centrifuged at 13,000 rpm for 15 min at 4 °C. The supernatant was injected directly into an Agilent triple-quad (QQQ, 6495) instrument operated in multiple reaction monitoring mode (MRM), where the collision energies (CE) and product ions (MS2 or quantifier and qualifier ion transitions) were pre-optimized. Transition of 365.3–267.1 (CE: 18 ev) was used as the quantifier, and other transitions including 365.3–253.1 (CE: 26 ev), 365.3–249.1 (CE: 22 ev), and 365.3–223.1(CE: 26 ev) were used as the qualifiers. Cycle time was 200 ms for each transition. ESI source conditions were set as follows: gas temperature 250 °C, gas flow 14 l/min, nebulizer 20 psi, sheath gas 250 °C, sheath gas flow 11 l/min, capillary voltage 3000 V, nozzle voltage 1500 V and EMV 200 V in ESI negative mode. Analyses were performed on a Poroshell 300SB-C18 column (length 75 mm × internal diameter 2 mm, particle size 5 mm). The mobile phase was composed of A = 0.1% formic acid in water and B = 0.1% formic acid in acetonitrile. A linear gradient from 30% B (0–2 min) to 100% B (2–5 min, maintained for 5 min) was applied. The flow rate was 300 ml/min, and the sample injection volume was 5 ml. Due to the lack of an isotope-labeled standard of KDT501, the recovery and matrix effect were evaluated using a matrix (e.g., liver) spiking method with different levels. Overall recovery of spiked KDT501 in tissue samples was between 90% and 105% (*n* = 3 for each spiking level), and the matrix effect was similar between tissues, denoting the accuracy and robustness of the analytical methods.

### 2.13 Activity-based protein profiling (ABPP)

To evaluate the ability of KDT501 to inhibit the serine hydrolase DPP-IV, we used activity-based protein profiling, a chemoproteomic method that allows measurement of the activity of enzymes in native systems using chemical probes. ABPP experiments were conducted as previously described [36]. Briefly, HEK 293T cell lysates overexpressing recombinant DPP-IV were incubated with KDT501 or KR-62436 (a DPP-IV inhibitor) for 30 min prior to the addition of a rhodamine-labeled fluorophosphonate probe that reacts broadly with many serine hydrolases, including DPP-IV. After 8 min, reactions were quenched by addition of 4X loading dye and boiling at 95 °C. Proteomes were separated by SDS-PAGE and DPP-IV activity (i.e. fluorescence) visualized on a ChemiDoc Imaging System (Bio-Rad).

### 2.14 Profile of KDT501 in bitter taste receptor panels

KDT501 was screened against 34 mouse and 25 human bitter taste receptors using a recombinant expression system as described previously [37], [38]. Briefly, plasmids encoding bitter taste receptor cDNAs were individually transfected into HEK293T cells stably expressing the chimeric G protein Gα16gust44. 24 h later, cells were loaded with Fluo-4 AM (Molecular Probes) in the presence of 2.5 mM probenecid (Sigma–Aldrich). Media was changed to 130 mM NaCl, 5 mM KCl, 10 mM HEPES, 2 mM CaCl_2_, 10 mM glucose, pH 7.4 prior to analysis of calcium release upon injection of KDT501 on a FLIPR plate reader (Molecular Devices). Peak responses were normalized relative to background fluorescence (ΔF/F = (F – F_0_)/F_0_).

### 2.15 Gene expression analysis

Total RNA was extracted using the Directzol RNA miniprep kit (Zymo Research) and analyzed by Taqman-based qRT-PCR (SuperScript III Platinum; Life Technologies) on an ABI 7900HT system (Applied Biosystems). Samples were analyzed as multiplexed reactions with 36B4 as internal control. Quantification was carried out using the standard curve method.

### 2.16 Statistical analysis

Statistical significance was assessed using two-way ANOVA with *post hoc* Bonferroni tests when comparing multiple treatments between two or more groups, one-way ANOVA with *post hoc* Tukey's tests when comparing multiple treatments within a single group, and Student's *t* test when comparing two treatments. The *n* value refers to the number of mice or biological replicates for cell-based assays as specified in figure legends. A *P* value < 0.05 was considered statistically significant and data were analyzed using GraphPad Prism 5.0 (GraphPad Software). Details of each statistical analysis are provided in the figure legends.

## 3 Results

### 3.1 KDT501 is a selective ligand of the bitter taste receptor Tas2r108

**Figure 1:**
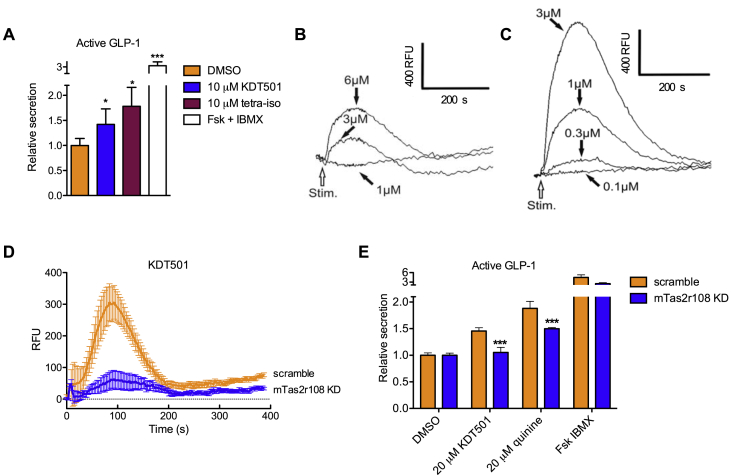
**KDT501 signals through the bitter taste receptor mTas2r108**. (A) Secretion of active GLP-1 into media from STC-1 cells exposed to the indicated compounds for 1 h. Forskolin (20 μM)/IBMX (100 μM) cocktail serves as positive control (n = 3). Representative intracellular calcium traces (n = 3) induced by KDT501 in HEK293 cells stably-expressing a Gα15-gustducin chimera and transiently transfected with (B) mouse Tas2r108 or (C) human TAS2R1. Calcium traces are shown after mock subtraction; RFU = relative fluorescence units. (D) Intracellular calcium mobilization response to KDT501 (10 μM) in STC-1 cells stably-expressing scramble shRNA or shRNA targeting Tas2r108. The response to KDT501 is abolished in cells depleted of Tas2r108 (n = 6). (E) Secretion of active GLP-1 in Tas2r108 knockdown STC-1 cells. Data presented as means ± SD in A and E, and means ± SEM in D. Statistical significance was calculated using Student's *t* test in A, and two-way ANOVA in E. *p < 0.05, ***p < 0.005 relative to vehicle-treated or Tas2r108 knockdown cells.

As a first step to discern the utility of targeting extraoral bitter taste receptors in metabolic disease, we tested the hypothesis that the beneficial effects of KDT501 and isohumulones are mediated by activation of Tas2rs by evaluating KDT501 in assays indicative of bitter taste receptor signaling. As reported for bitter tastants, KDT501 was able to stimulate secretion of active GLP-1 in cultured STC-1 enteroendocrine cells (Figure 1A). This property was shared by a natural tetrahydroisohumulone mixture (Figure 1A, Supplementary Fig. 1A). To determine the extent to which this effect was mediated by bitter taste signaling, we profiled KDT501 against 34 of 35 mouse and all 25 human bitter taste receptors using a heterologous system and measuring intracellular calcium mobilization [37], [38]. We found that KDT501 signaled through a single mouse bitter taste receptor, Tas2r108 (Figure 1B, Supplementary Fig. 1B). Similarly, KDT501 acted as a specific agonist for a single human receptor, TAS2R1 (Figure 1C, Supplementary Fig. 1B). Knockdown of Tas2r108 in STC-1 cells ablated the ability of KDT501 to stimulate intracellular calcium transients (Figure 1D, Supplementary Fig. 1D). These cells also failed to respond to emetine and quinine, known Tas2r108 agonists [37] (Supplementary Fig. 1C,D). Furthermore, the ability of STC-1 cells depleted of Tas2r108 to secrete active GLP-1 in response to KDT501 was lost (Figure 1E). These findings support the notion that this isohumulone analog signals through the bitter taste receptor Tas2r108. Given that many bitter tastants signify toxicity, perhaps due to pleiotropic effects, and that, in contrast, this pure, synthetic non-racemic isohumulone derivative is well tolerated in rodents and humans [13], [39], we reasoned that KDT501 could be a useful tool to ascertain the benefits of selectively activating bitter taste signaling in metabolic disease.

### 3.2 KDT501 is a GLP-1 secretagogue

**Figure 2:**
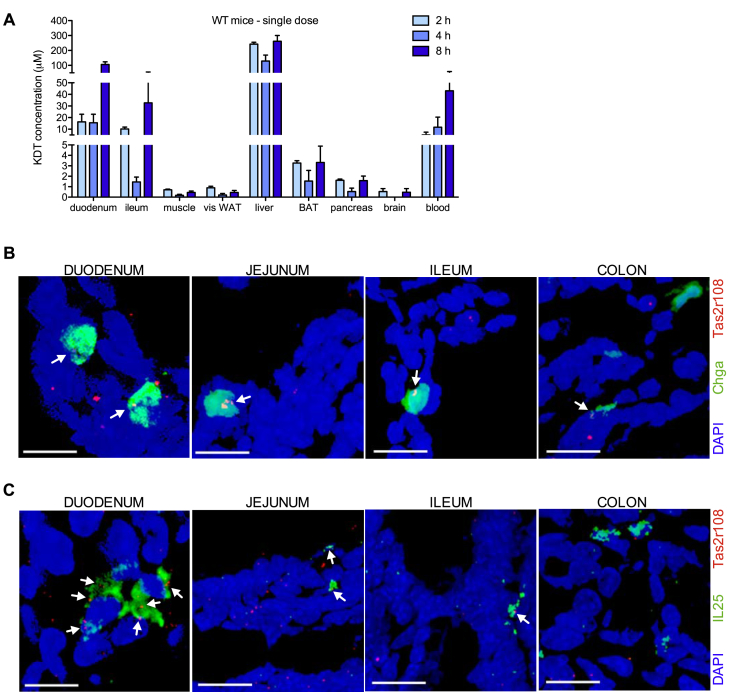
**Tissue distribution of KDT501 and its cognate receptor *Tas2r108***. (A) Tissue distribution of KDT501 in wild type mice gavaged with a single oral dose (150 mg/kg) of KDT501 (n = 3). RNAscope images showing co-localization (indicated by white arrows) of *Tas2r108* (red signal) and (B) enteroendocrine (*Chga*; green signal) or (C) tuft cell markers (*Il25*; green signal) throughout the gastrointestinal tract. Scale bars are 10 μm.

**Figure 3:**
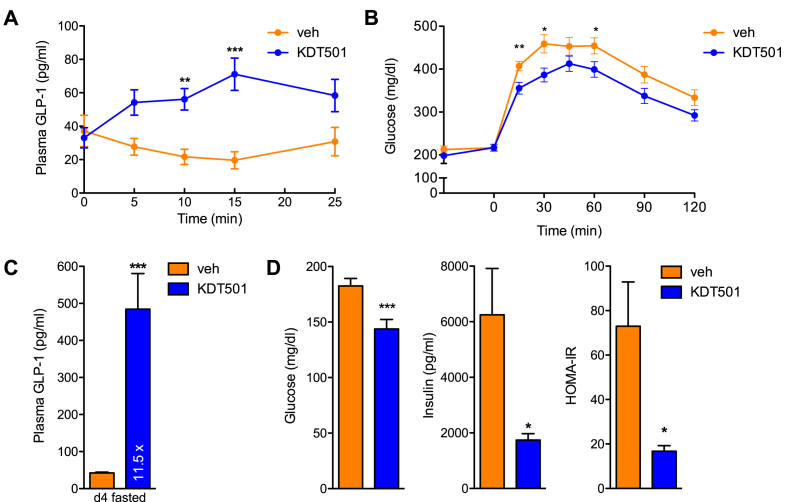
**KDT501 acts as a GLP-1 secretagogue to improve glucose homeostasis**. (A) Effect of a single oral dose of KDT501 on GLP-1 circulating levels in naïve DIO mice (n ≥ 7). (B) Glucose tolerance test in naïve DIO mice after a single dose of KDT501 administered 30 min prior to an oral glucose bolus (n ≥ 7). (C) Plasma GLP-1 levels in DIO mice treated with 150 mg/kg KDT501 for 4 days and dosed 30 min prior to blood collection in tubes containing a DPP-IV inhibitor (n ≥ 8). (D) Plasma glucose and insulin levels, and HOMA-IR in fasted DIO mice after 4 days of 150 mg/kg KDT501 treatment (n ≥ 8). Data presented as means ± SEM. Statistical significance was calculated using two-way ANOVA in A–B, and Student's *t* test in C–D. *p < 0.05, **p < 0.01, ***p < 0.001 relative to vehicle-treated mice.

Characterization of the effects of KDT501 in rodents has been very limited [13]. The tissues at which KDT501 acts to enhance insulin sensitivity, and its mode of action, are not known. Thus, to better interpret any effects we might uncover in mice chronically-treated with KDT501, we performed pharmacokinetic studies to assess exposure. Tissue distribution in wild type mice after a single oral dose (150 mg/kg) was limited to liver and the gastrointestinal tract, with low or negligible levels of KDT501 found in adipose tissue and none in brain (Figure 2A). Expression of bitter taste receptors in the gastrointestinal tract is not as extensive as in the oral cavity [19], [26]. As assessed by quantitative real-time PCR, only five Tas2rs are expressed throughout the entire mouse gastrointestinal tract (Tas2r108, 126, 135, 137, and 143), with Tas2r108 being the most abundant [40]. To further define the intestinal cell types in which Tas2r108 is expressed, we performed RNAscope studies [41] and found that Tas2r108 is expressed throughout the intestine, including in enteroendocrine (secretory vesicle protein chromogranin A, *Chga*, positive) and tuft (*Il25* positive) cells (Figure 2B,C and Supplementary Fig. 2). Because enteroendocrine cells are responsible for GLP-1 secretion, we next determined if a single dose of KDT501 could induce intestinal GLP-1 release in the context of metabolic dysfunction. We treated fasted DIO mice with a single oral dose of KDT501 (150 mg/kg) and collected blood in the presence of a DPP-IV inhibitor to prevent GLP-1 degradation. KDT501 administration increased plasma GLP-1 levels 3-fold within 15 min of oral gavage (Figure 3A). Further, pre-treatment of naïve DIO mice with a single oral dose of KDT501 30 min prior to an oral glucose challenge considerably improved glucose clearance (Figure 3B), indicating that this compound is indeed an incretin secretagogue. To assess the extent to which this beneficial effect would be sustained over time, we treated a second cohort of DIO mice with multiple doses (150 mg/kg daily). Four days of oral KDT501 treatment resulted in a >10-fold surge in GLP-1 levels that was accompanied by rapid improvement in insulin sensitivity (Figure 3C,D). Importantly, KDT501 is not a DPP-IV inhibitor (Supplementary Fig. 3), indicating that direct Tas2r108 activation likely mediates the GLP-1 secretagogue effect of KDT501.

### 3.3 KDT501 treatment ameliorates obesity-diabetes

**Figure 4:**
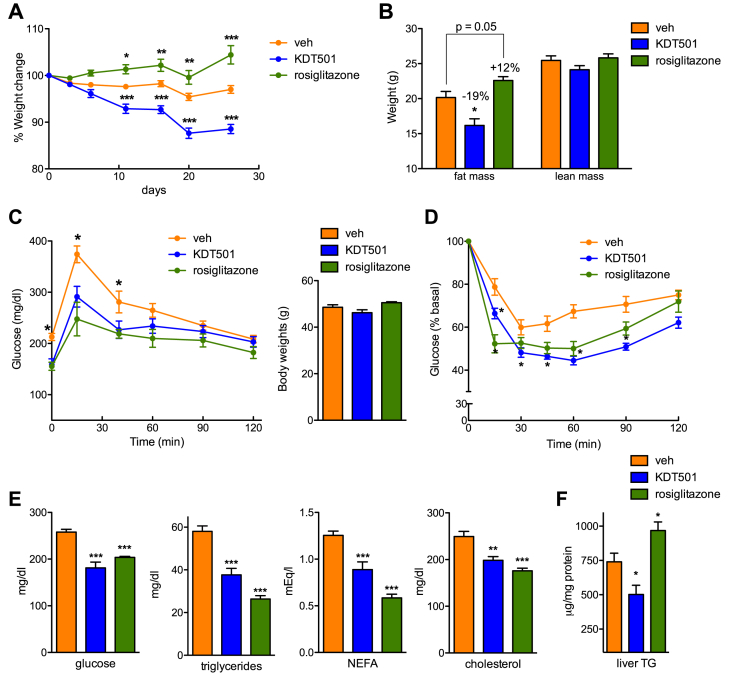
**Chronic KDT501 treatment elicits loss of fat mass and improves plasma lipids**. (A and B) Evolution of body weight (A), and final body composition (B) of DIO mice treated with vehicle, 150 mg/kg KDT501, or 10 mg/kg rosiglitazone for 28 days (n ≥ 8). (C) Oral glucose tolerance test in DIO mice treated for 7 days with vehicle, KDT501, or rosiglitazone. Body weights (right panel) were not different at this time (n ≥ 8). (D) Insulin tolerance test after 17 days of treatment (n ≥ 8). (E) Fasted glycemia and plasma lipid profile (n ≥ 7). (F) Hepatic triglyceride (TG) content (n ≥ 7). Data presented as means ± SEM. Statistical significance was calculated using two-way analysis of variance (ANOVA) in A-D, one-way ANOVA in E, and Student's *t* test in F. *p < 0.05, **p < 0.01, ***p < 0.001 relative to vehicle-treated mice.

Given the ability of KDT501 to increase endogenous GLP-1 levels, we next examined the effects in DIO mice of chronic treatment with this selective bitter taste receptor activator. Intragastric KDT501 administration (150 mg/kg daily) for 28 days resulted in considerable improvement of many features of metabolic syndrome. Treated mice lost weight within 11 days of the start of treatment, a loss that could be ascribed to decreased fat mass (Figure 4A,B). In contrast, mice treated with rosiglitazone (4 mg/kg), a synthetic PPARγ ligand used here as a control for enhanced insulin sensitivity and adipose tissue beiging, gained weight and fat mass (Figure 4A,B). Changes in body weight were not due to differences in food intake (Supplementary Fig. 4A). Analysis of glucose homeostasis parameters revealed that KDT501-treated DIO mice displayed improved glucose tolerance *prior* to significant changes in body weight (Figure 4C), corroborating our findings in acute studies indicating that KDT501 behaves as an incretin secretagogue. Treatment also increased insulin sensitivity, with KDT501 eliciting comparable improvements to those seen with the clinical insulin sensitizer rosiglitazone (Figure 4D). Loss of adipose tissue mass in KDT501-treated mice was reflected in reduced lipid accumulation in brown adipose tissue (Supplementary Fig. 4B). Adipocyte size distribution in subcutaneous and visceral white fat depots was not significantly altered (Supplementary Fig. 4C), but the weight of all adipose depots was reduced (Supplementary Fig. 4D). Plasma analysis showed notable improvement in glucose and lipid profiles, comparable to that brought about by the clinical agent rosiglitazone. Mice treated with KDT501 had lower levels of non-esterified free fatty acids (NEFA), triglycerides (TGs), total cholesterol, and fasting glucose (Figure 4E). KDT501 treatment also reduced liver triglyceride accretion, in contrast to the effect of rosiglitazone that increased liver lipid accumulation, a side effect of thiazolidinediones in rodents [42] (Figure 4F and Supplementary Fig. 4B). In addition, treatment with KDT501 increased circulating ketone bodies (Supplementary Fig. 4E), though no changes were seen in expression of genes involved in ketogenesis or fatty acid oxidation in the liver (Supplementary Fig. 5A,B). A reduction in circulating ALT and AST levels, likely reflecting the lower levels of steatosis, was also seen with KDT501 dosing, indicating that treatment is safe (Supplementary Fig. 4F). Interestingly, KDT501 treatment reduced expression of genes involved in hepatic lipogenesis, lipid uptake, and gluconeogenesis, while increasing *Fgf21* levels (Supplementary Fig. 5C,D). The benefits of KDT501 treatment were accompanied by a strikingly broad suppression of circulating levels of multiple proinflammatory cytokines and chemokines that are increased in obese-diabetic mice (Supplementary Fig. 6).

**Figure 5:**
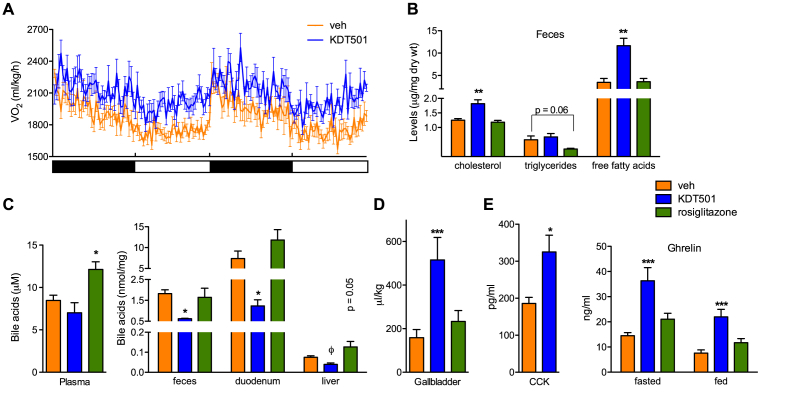
**KDT501 increases energy expenditure and limits intestinal lipid absorption**. (A) Oxygen consumption rate in vehicle or KDT501-treated DIO mice over two light-dark cycles (n = 7). (B) Fecal lipid content after 25 days of treatment (n = 4). (C) Plasma, fecal, and tissue bile acid levels (n ≥ 4). (D) Gallbladder volume in a separate cohort of DIO mice treated for 15 days (n ≥ 5). (E) CCK and ghrelin levels in KDT501-treated DIO mice (n ≥ 8). Data presented as means ± SEM. Statistical significance was calculated using one-way ANOVA (*p < 0.05, **p < 0.01, ***p < 0.001) in B–E, and Student's *t* test (^ϕ^p < 0.05) in C relative to vehicle-treated mice.

To examine the basis of the weight loss seen upon long-term KDT501 administration, we measured energy balance parameters. Mice treated with KDT501 showed increased energy expenditure, particularly in the light phase, but no difference in locomotor activity (Figure 5A, Supplementary Fig. 7A,B). KDT501-treated mice displayed increased heat production and a trend towards reduced respiratory exchange ratio, indicative of greater utilization of lipids as fuel (Supplementary Fig. 7C,D). To assess if the increase in energy expenditure brought about by KDT501 treatment could be due to beiging/browning of white adipose tissue or enhanced brown fat activity, we analyzed gene expression in these depots. In spite of the pronounced effect of KDT501 on energy expenditure, no changes in expression of thermogenic, oxidative, or lipid handling genes were observed in white or brown adipose tissue (Supplementary Fig. 8). As expected, rosiglitazone treatment did stimulate oxidative gene expression in white adipose depots (Supplementary Fig. 8). To complete the study of energy balance parameters, we analyzed intestinal nutrient absorption. KDT501 treatment resulted in increased levels of free fatty acids and cholesterol present in feces (Figure 5B). Because bile acids are key determinants of intestinal lipid absorption, we hypothesized that KDT501 treatment may have led to alterations in bile acid homeostasis. Indeed, bile acid levels in intestine, liver, and feces were reduced, while sequestration of bile acids in the gallbladder was increased (Figure 5C,D). Paradoxically, circulating levels of cholecystokinin (CCK), a principal regulator of gallbladder contraction and bile acid release, were higher in KDT501-treated mice (Figure 5E). Similarly, levels of the ‘hunger’ hormone ghrelin were elevated in KDT501-treated mice in both fed and fasted states (Figure 5E). Moreover, no changes in expression of genes involved in hepatic and intestinal bile acid synthesis or transport (e.g., Cyp7a1, FXR, BSEP) were noted. However, expression of markers of bile acid signaling in the intestine (e.g., SHP, Gpbar1 also known as TGR5) was noticeably reduced (Supplementary Fig. 9A,B). Interestingly, expression of LXRα and all its targets (e.g., ABCA1, ABCG8) was decreased in the intestine, and SREBP2 levels were increased in the intestine as well as the liver (Supplementary Fig. 9A,B). These findings, suppressed expression of intestinal sterol efflux genes and increased expression of hepatic sterol synthesis regulators, suggest that reduced absorption of sterols in KDT501-treated mice results in feedback regulation to maintain tissue sterol content. In sum, the weight loss seen in KDT501-treated mice was the result of enhanced energy expenditure and reduced lipid absorption.

### 3.4 DPP-IV inhibition potentiates the GLP-1 secretagogue effect of KDT501

**Figure 6:**
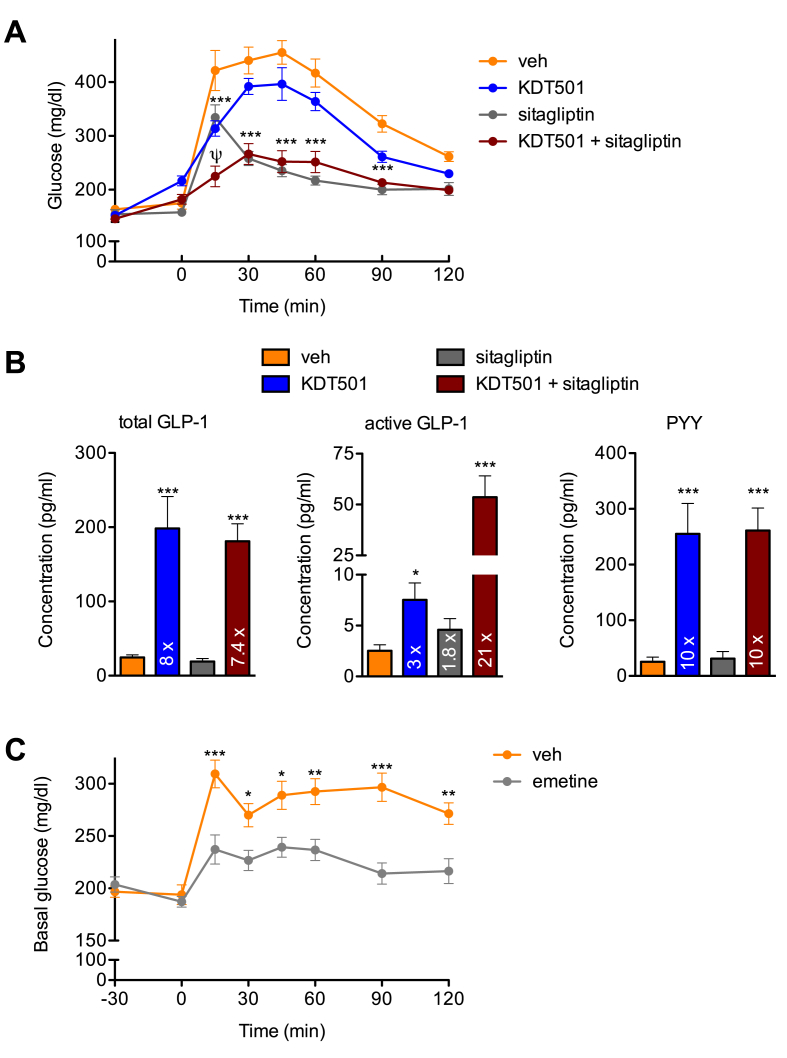
**Co-treatment with a DPP-IV inhibitor enhances the response to KDT501**. (A) Glucose tolerance test in naïve DIO mice after a single dose of vehicle, 150 mg/kg KDT501, 4 mg/kg sitagliptin (DPP-IV inhibitor), or the combination administered 30 min prior to an oral glucose bolus (n ≥ 6). (B) Total GLP-1, active GLP-1, and PYY levels in plasma of DIO mice treated for 4 days and dosed 30 min prior to blood collection in tubes containing a DPP-IV inhibitor (n ≥ 6). (C) Glucose tolerance test in naïve DIO mice after a single dose of vehicle, or 30 mg/kg emetine (mTas2r108 and 140 agonist) administered 30 min prior to an oral glucose bolus (n ≥ 9). Statistical significance was calculated using two-way analysis of variance (ANOVA) in A and C, and one-way ANOVA in B. ^ψ^p < 0.01 versus all other groups, *p < 0.05, **p < 0.01, ***p < 0.001 relative to vehicle-treated mice.

To further validate the translational potential of a bitter taste receptor agonist such as KDT501 to modulate gut hormone secretion, particularly its stimulation of endogenous GLP-1 release, we pre-treated naïve DIO mice with a single oral dose of KDT501, a DPP-IV inhibitor in clinical use (sitagliptin), or the combination 30 min prior to an oral glucose challenge. DIO mice treated with KDT501 or sitagliptin both showed improved glucose tolerance, and the combination had a striking additive effect (Figure 6A). Further, DIO mice dosed with KDT501 for four days, but not those treated only with sitagliptin, showed an ∼8-fold increase in total circulating GLP-1 (Figure 6B). These same mice had a 3-fold increase in active GLP-1, while mice treated only with sitagliptin showed a 1.8-fold boost (Figure 6B). Notably, DIO mice treated with the combination of KDT501 and sitagliptin had a dramatic 21-fold increase in active GLP-1 levels (Figure 6B), highlighting the potential of combining an incretin secretagogue (KDT501) and a DPP-IV inhibitor to achieve greater glycemic control. PYY was the only other gut hormone tested that showed a significant increase (∼10-fold) in these KDT501-treated, fasted mice (Figure 6B). CCK levels showed a trend to increase that was not significant, while levels of active ghrelin were unchanged (Supplementary Fig. 10). These observations suggest that the ability of KDT501 to alter CCK and ghrelin levels is more progressive and may require longer dosing than its effect on GLP-1 and PYY.

Finally, to determine if the incretin-secretagogue properties of KDT501 in mice were shared by other bitter tastants, we assessed the acute effects of another Tas2r108 agonist, emetine, in DIO mice. Pre-treatment with a single oral dose of emetine 30 min prior to an oral glucose bolus markedly enhanced glucose tolerance in DIO mice (Figure 6C). In aggregate, these findings indicate that that selective targeting of intestinal Tas2r108, and perhaps other bitter taste receptors with a similar expression pattern, can be of benefit in metabolic disease.

## 4 Discussion

Synthetic modification of natural products has resulted in the development of numerous therapeutic agents [43]. This success reflects the intrinsic properties of natural bioactive molecules. They are inherently more bioavailable than novel chemical matter, and their effects are often the result of simultaneous modulation of several biological processes. This last feature endows natural compound derivatives with a double-edged advantage. They can effectively target multiple features of complex diseases such as metabolic syndrome, but understanding their mode of action and their molecular target(s) is hindered by their pleiotropic nature. Metformin, a derivative of isoamylene guanidine present in French lilac [44], exemplifies this dichotomy. It has been used as first-line therapy for diabetes for decades, yet its mode of action and relevant targets remain subjects of debate [45], [46], [47]. Here, we have used KDT501, a pure analog of hops-derived isohumulones, to identify the molecular mechanisms that contribute to the benefits seen in metabolic syndrome with natural mixtures. We have found that one way whereby KDT501 elicits multifaceted improvements in DIO mice is via modulation of a primary molecular target, the bitter taste receptor Tas2r108. Using this pure, selective Tas2r108 agonist to systematically profile the impact of chronic extraoral bitter taste receptor agonism in a model of obesity and insulin resistance, we have demonstrated the utility of targeting bitter taste receptors in metabolic disease.

Bitter taste receptors provide the sensory capacity to detect bitterness in food [17], [21]. These GPCRs are thought to have evolved as a chemosensory mechanism to prevent the ingestion of poisons, which are often bitter. While they were originally identified in the tongue, it is now clear that bitter taste receptors and their signaling components are broadly expressed in extraoral tissues, including airway epithelia, the gastrointestinal tract, the immune system, brain, liver, kidney, skin, and reproductive tissues [22], [23], [48]. The impact on systemic physiology of bitter taste signaling in these tissues has only recently begun to emerge. For example, activation of bitter taste receptors expressed in airway epithelia increases the beating frequency of cilia in these cells, ostensibly to clear out noxious agents [49]. The relevance of extraoral bitter taste signaling is reflected by the finding that polymorphisms in human TAS2Rs have been linked to varied (patho)physiologic processes, including resistance to bacterial infections [50], development of cardiovascular disease [51], and even cancer [52]. TAS2R variants have also been linked to human metabolic dysfunction. For instance, defective TAS2R9 signaling is associated with altered glucose and insulin homeostasis [29].

Tas2r isoforms are differentially expressed throughout the entire gastrointestinal tract, from stomach to colon [40]. Tas2r108 is one of 5 receptors (of 35) that are expressed in all these regions. Gastrointestinal bitter taste signaling has been shown to have effects on intestinal motility, gastric emptying and satiety [53], [54], [55], ion and fluid secretion [56], [57], expression of efflux transporters [58], and secretion of enteroendocrine hormones, including ghrelin [54], CCK [25], [26], [27], and GLP-1 [26], [29]. Chronic KDT501 treatment increased plasma levels of all these hormones. We focused our analysis on GLP-1 because increased GLP-1 action is an established mechanism [59] that can explain the rapid improvement in glucose homeostasis we noted in KDT501-treated DIO mice prior to weight loss. Several studies have shown that bitter tastants can induce secretion of GLP-1 *in vitro* [26], [29], and one has reported increased GLP-1 levels in mice treated acutely with bitter taste receptor agonists [28]. However, whether the magnitude of this stimulation could impact systemic physiology, and the extent to which the increase in circulating GLP-1 levels could be of utility in established disease had not been tested [30]. Here, using a pure compound that activates a single Tas2r, we provide proof-of-principle that agonism of intestinal bitter taste receptors can be therapeutic in metabolic disease. Agents such as KDT501 that stimulate endogenous GLP-1 secretion may provide an attractive alternative to GLP-1 mimetics and DPP-IV inhibitors in clinical use. Our data indicates that they could also be used together with DPP-IV inhibitors, for the combination elicits drastically higher levels of active GLP-1 than either agent alone.

The ability of KDT501 to stimulate incretin secretion can account for the enhancement of glucose homeostasis in the absence of weight loss, but the loss of fat mass seen with sustained treatment undoubtedly contributed to the ensuing improvement of other features of metabolic syndrome, including dyslipidemia, hepatic steatosis, and chronic inflammation. KDT501 treatment increased energy expenditure in the absence of changes in activity. This effect could be related to the ability of KDT501 to induce GLP-1 secretion, for there are reports that GLP-1 itself can promote energy expenditure in rodents [60], [61], [62], [63] and humans [64]. It is also possible that the increase in hepatic *Fgf21* expression seen in mice treated chronically with KDT501 may have contributed to the enhancement of energy expenditure and glucose homeostasis.

KDT501 treatment also profoundly altered bile acid cycling and decreased intestinal lipid absorption, an effect that likely added to the weight loss seen and the long-term benefits of treatment. KDT501 appears to modulate bile acid cycling by prompting sequestration of bile acids in the gallbladder. Because CCK secretion in response to food results in the release of bile acids from the gallbladder, we surmised that KDT501 might be acting to decrease CCK signaling. Surprisingly, we found that CCK levels were elevated in fasted KDT501-treated mice. Moreover, KDT501 is not a CCK receptor antagonist [13]. It is possible that increased CCK levels reflect a physiologic feedback mechanism to compensate for the decrease in bile acids. Alternatively, the ability of KDT501 to activate Tas2r108 may provide a basis for these findings. Studies have shown that activation of bitter taste receptors in enteroendocrine cells and in mice stimulates CCK release [26], [27]. It is also possible that KDT501-induced sequestration of bile acids in the gall bladder may be due to increased GLP-2 levels. This proglucagon-derived peptide is co-secreted with GLP-1 in response to nutrients and has been recently shown to regulate gallbladder refilling [65] In addition to its effects on satiety, gastric motility, and release of bile acids and pancreatic enzymes, intestinal CCK stimulated by bitter taste agonism acts in a paracrine manner to induce expression of the efflux transporter ABCB1 in enterocytes to avoid uptake of bitter-tasting toxins [58].Our results also present an interesting dichotomy, whereby decreased nutrient absorption is not balanced by an increase in food intake. This observation reflects a complex interplay in KDT501-treated mice between elevated GLP-1, CCK, and PYY levels (which promote satiety), and increased ghrelin levels (which induce feeding). It has been shown that bitter taste receptor agonists induce ghrelin secretion in mice [54]. The benefits we report in KDT501-treated mice are thus likely the sum of increased GLP-1 secretion and a variety of GLP-1 independent effects. Further work using Tas2r108 null strains is required to understand how KDT501, and more generally intestinal bitter taste receptor signaling, remodels bile acid metabolism and the full range of enteroendocrine hormones it regulates.

In addition to its effects on metabolic parameters, KDT501 treatment resulted in a dramatic reduction of multiple proinflammatory cytokines and chemokines. Because chronic low-grade inflammation exacerbates metabolic dysfunction [66], it is likely that the anti-inflammatory action of KDT501 contributed to the metabolic benefits seen. We cannot determine, however, the extent to which KDT501-mediated suppression of inflammation is a cause rather than a reflection of the improved metabolic state. Natural isohumulone mixtures have anti-inflammatory properties that may be related to inhibition of NF-κB signaling [67], [68]. We note, however, that bitter taste signaling plays a prominent role in the defense against pathogens [48]. For example, activation of bitter taste receptors in airway epithelia and solitary chemosensory cells by bacteria-derived quorum sensing molecules results in secretion of antimicrobial peptides and production of nitric oxide to kill bacteria and prevent biofilm formation [50], [69]. In the gut, activation of taste receptors in tuft cells initiates the type 2 immune response against parasites [70], [71], [72]. In addition, expression of multiple TAS2Rs has been detected in subpopulations of circulating leukocytes [73]. Tas2r108 itself is expressed in blood cells [74] and the concentration of KDT501 in blood is sufficient to activate it. Further, a mixture of tetrahydroisohumulones enhanced gut barrier function and protected mice from high-fat diet-induced metabolic endotoxemia [8]. Thus, it is possible that the anti-inflammatory properties of KDT501 are due to bitter taste agonism. More studies will be required to examine this notion.

We have demonstrated that specific modulation of bitter taste receptor signaling in extraoral tissues improves multiple features of metabolic disease. In the gut, bitter taste agonism alters enteroendocrine hormone secretion in a pattern reminiscent of that seen after bariatric surgery [75]. The broad effects of KDT501 intimate that bitter taste agonists may be useful in other conditions characterized by insulin resistance, dyslipidemia, and inflammation, such as non-alcoholic steatohepatitis and polycystic ovarian syndrome. In closing, we note that we also show that natural isohumulones also behave as GLP-1 secretagogues *in vitro*. Isohumulones are present in beer at 10–100 mg/L (∼30–300 μM) [11] and they activate human TAS2R1 and TAS2R14 [76]. Thus, it is possible that our findings may offer a partial basis for the widely debated benefits of beer consumption.
