# Supplementary material for: Intestinal bitter taste receptor activation alters hormone secretion and imparts metabolic benefits
Source: Mol Metab. 2018 Aug 4;16:76–87. doi: 10.1016/j.molmet.2018.07.013 (PMC6158035; doi:10.1016/j.molmet.2018.07.013)
Supplement: Multimedia component 1 [file mmc1.pdf]

SUPPLEMENTARY FIGURES

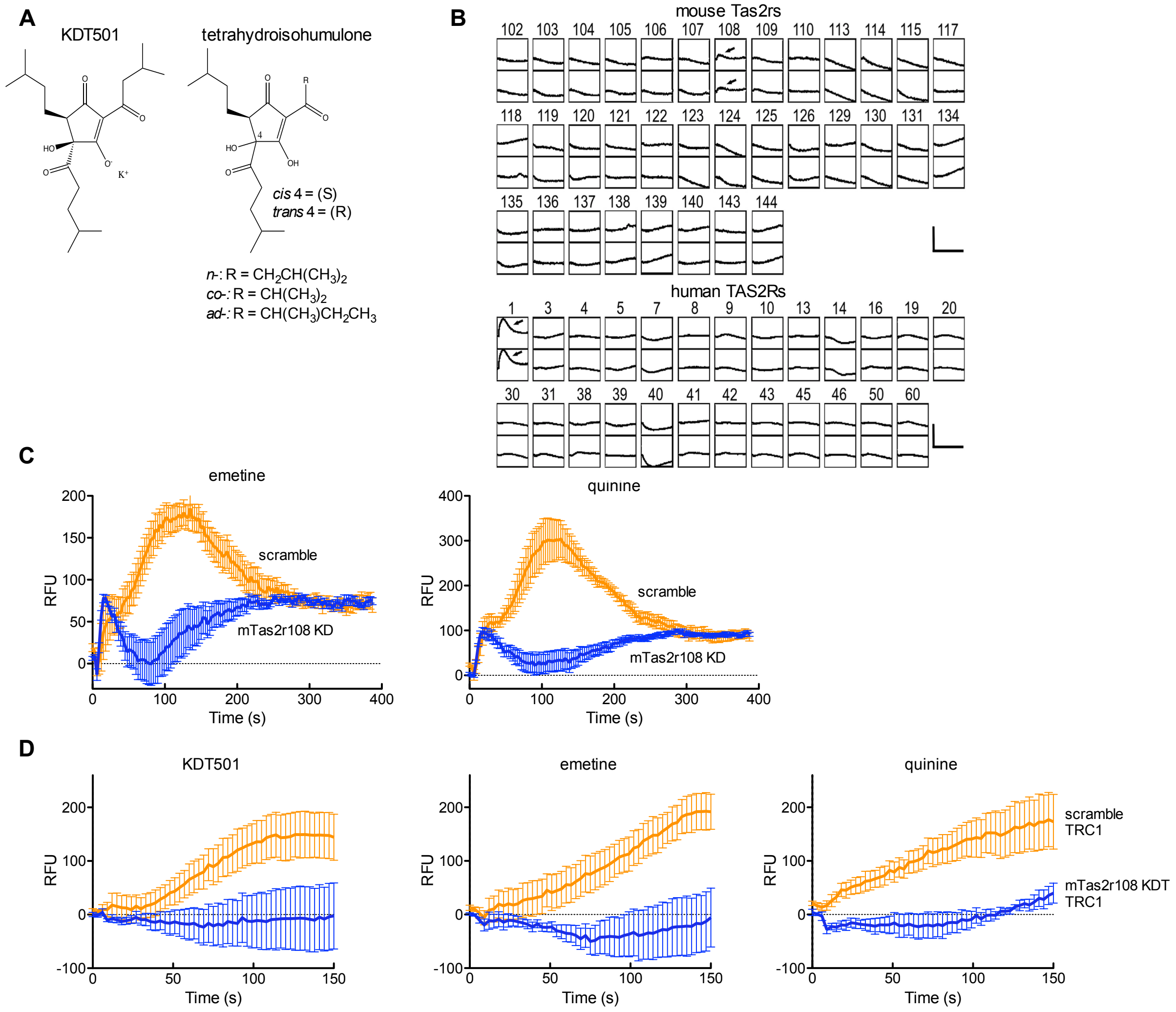

Figure S1. **KDT501 is a selective agonist of mouse Tas2r108 and human TAS2R1**.
(A) Chemical structures of KDT501 and the tetrahydroisohumulone mixture used in this study.
(B) Representative intracellular calcium traces (n = 3) of the response of 34 recombinant mouse and 25 human bitter taste receptors to 10 μM KDT501 tested using HEK293 cells stably-expressing a Gα15-gustducin chimera and transiently transfected with the indicated receptor. Calcium traces are shown after mock subtraction. Specific signals are denoted by arrows. Scale bars: y-axis = 3000 relative fluorescence units (RFU); x-axis = 300 s.
(C) Intracellular calcium mobilization response to Tas2r108 agonists (30 μM emetine and 20 μM quinine) in STC-1 cells expressing scramble lentiviral shRNA or targeting endogenous Tas2r108 using a second generation (TRC2) vector as shown in the main figures. The response to these known Tas2r108 agonists is abolished in cells depleted of Tas2r108 (n = 4).
(D) Intracellular calcium mobilization response to KDT501 (10 μM) and known Tas2r108 agonists (30 μM emetine and 20 μM quinine) in STC-1 cells expressing scramble lentiviral shRNA or targeting endogenous Tas2r108 using a distinct first generation (TRC1) vector (n = 4). Data are presented as means $\pm$ SEM.

A *Chga* co-localization

Tas2r108 positive
non-Tas2r108

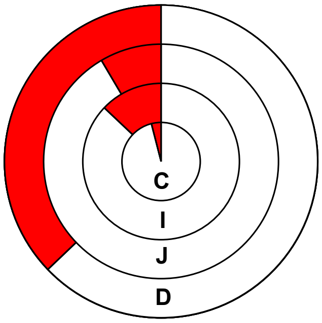

	duodenum	jejunum	ileum	colon
Tas2r108 positive	37.1	8.5	12.9	4.0
non-Tas2r108	62.9	91.5	87.1	96.0

B *Il25* co-localization

Tas2r108 positive
non-Tas2r108

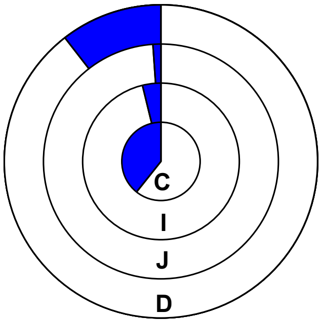

	duodenum	jejunum	ileum	colon
Tas2r108 positive	10.5	1.1	3.8	39.3
non-Tas2r108	89.5	98.9	96.2	60.7

C *Tas2r108* co-localization

EEC co-localization
Tuft co-localization
non-EEC or Tuft

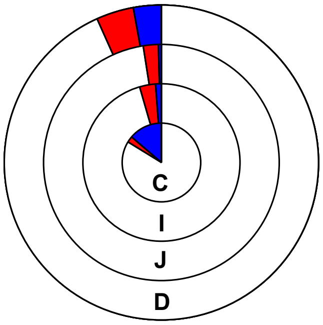

	duodenum	jejunum	ileum	colon
EEC co-localization	3.9	2.1	3.3	2.5
Tuft co-localization	2.9	0.4	1.2	13.8
other	93.3	97.5	95.5	83.8

Figure S2. RNAscope analysis of Tas2r108 expression in the intestine and co-localization with enteroendocrine and tuft cell markers.
(A and B) Percentage of (A) enteroendocrine cells (chromogranin A positive), and (B) Tuft cells (IL-25 positive) that express Tas2r108. D = duodenum; J = jejunum; I = ileum; C = colon.
(C) Percentage of Tas2r108 positive cells that express enteroendocrine or Tuft cell markers. Analysis was performed on three 200 μm x 200 μm sections. At least 60 chromogranin A, 90 IL-25, and 240 Tas2r108 positive cells were counted for each small intestine region. 50 chromogranin A, 28 IL-25, and 80 Tas2r108 positive cells were counted in colon sections.

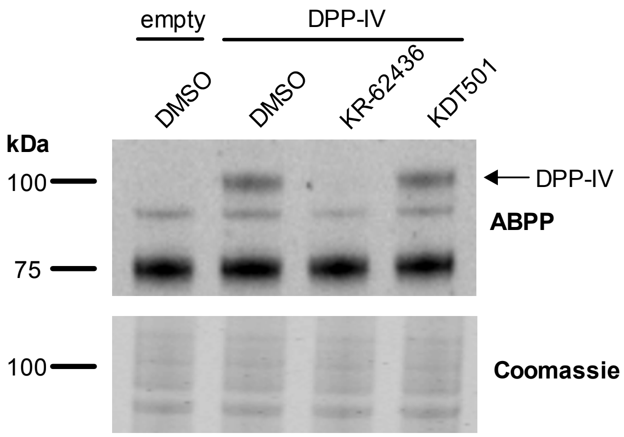

Figure S3. **KDT501 does not inhibit dipeptide peptidase IV (DPP-IV)**. Competitive gel-based activity-based protein profiling analysis of HEK 293T lysates overexpressing DPP-IV. Proteomes were incubated with DMSO, 5 μM KR-62436, or 75 μM KDT501 prior to the addition of a rhodamine-labeled fluorophosphonate probe (*top panel*). Protein loading was verified by Coomassie staining (*bottom panel*).

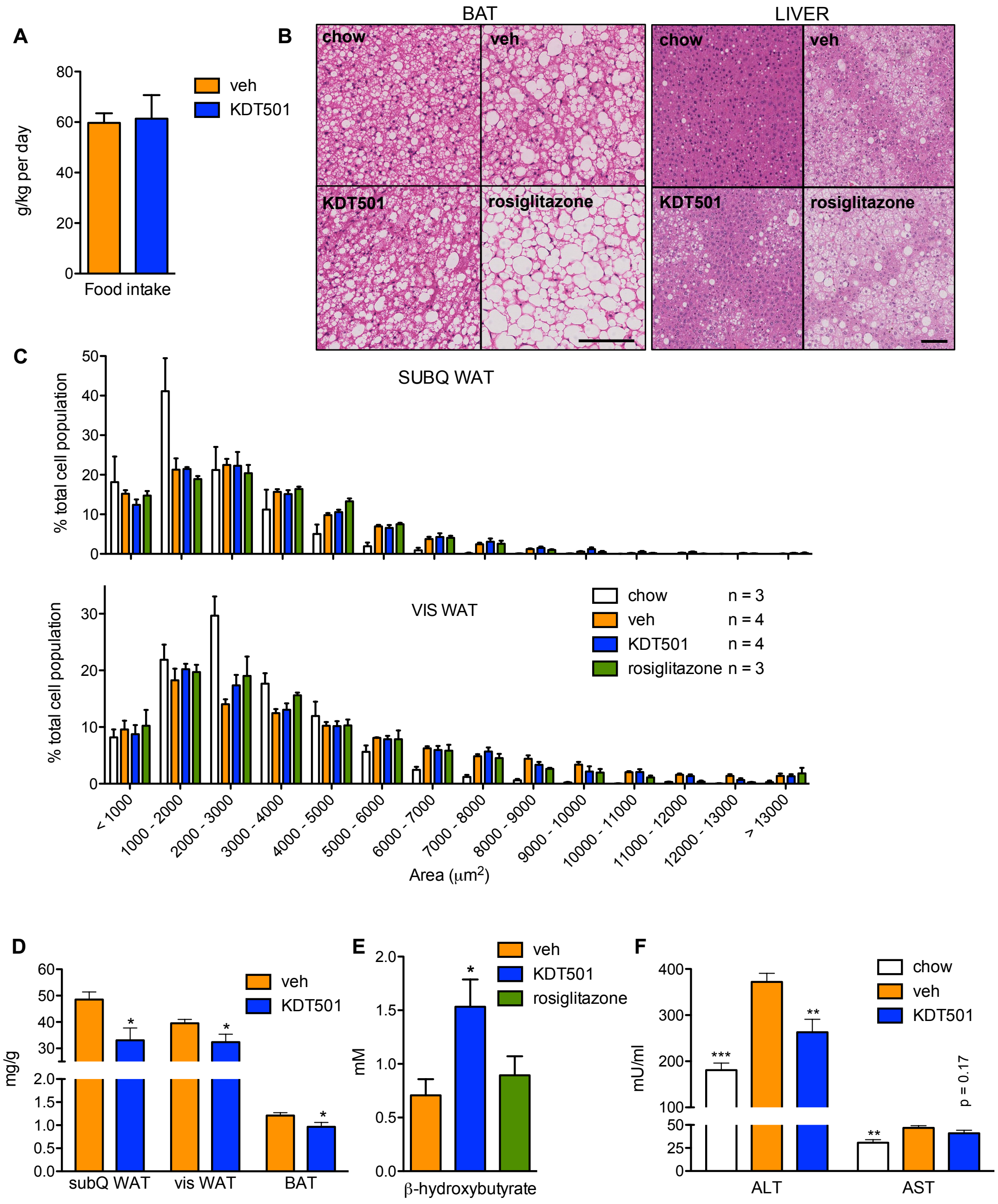

Figure S4. **Effect of KDT501 treatment on food intake, BAT and liver morphology, adipocyte size, plasma ketone bodies, and liver enzymes**.

(A) Food intake in CLAMS (comprehensive lab animal monitoring system) cages (n = 7).

(B) Representative brown adipose tissue (BAT) and liver H&E sections from DIO mice treated with vehicle, KDT501, or rosiglitazone, and matched normal chow controls. Scale bar = 100 μm.

(C) Adipocyte size distribution (subQ: subcutaneous, vis: visceral) from the mice shown in Figure 4 and matched chow controls (n as shown), and (D) adipose depot weights normalized to body weights (n = 6-8).

(E and F) Plasma β-hydroxybutyrate (E), and ALT and AST (F) levels in treated DIO mice (n ≥ 6). Data are presented as means ± SEM. Statistical significance was calculated using one-way ANOVA in D and E. *p < 0.05, **p < 0.01, ***p < 0.001 relative to vehicle-treated mice.

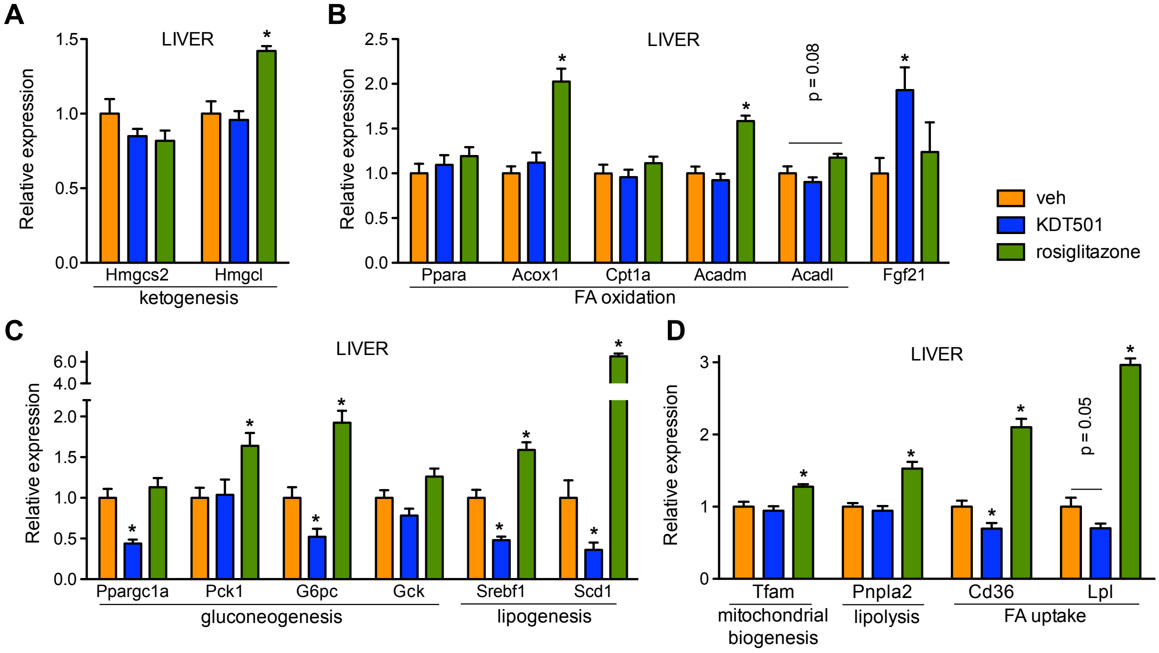

Figure S5. **Changes in hepatic gene expression elicited by KDT501 treatment**.
(A to D) Liver mRNA levels of genes involved in ketogenesis (A), fatty acid (FA) oxidation (B), gluconeogenesis, lipogenesis (C), and mitochondrial biogenesis, lipolysis, and fatty acid uptake (D) in DIO mice treated with vehicle, KDT501, or rosiglitazone ($n \geq 7$)
Data are presented as means ± SEM. Statistical significance was calculated using one-way ANOVA. *$p < 0.05$ relative to vehicle-treated mice.

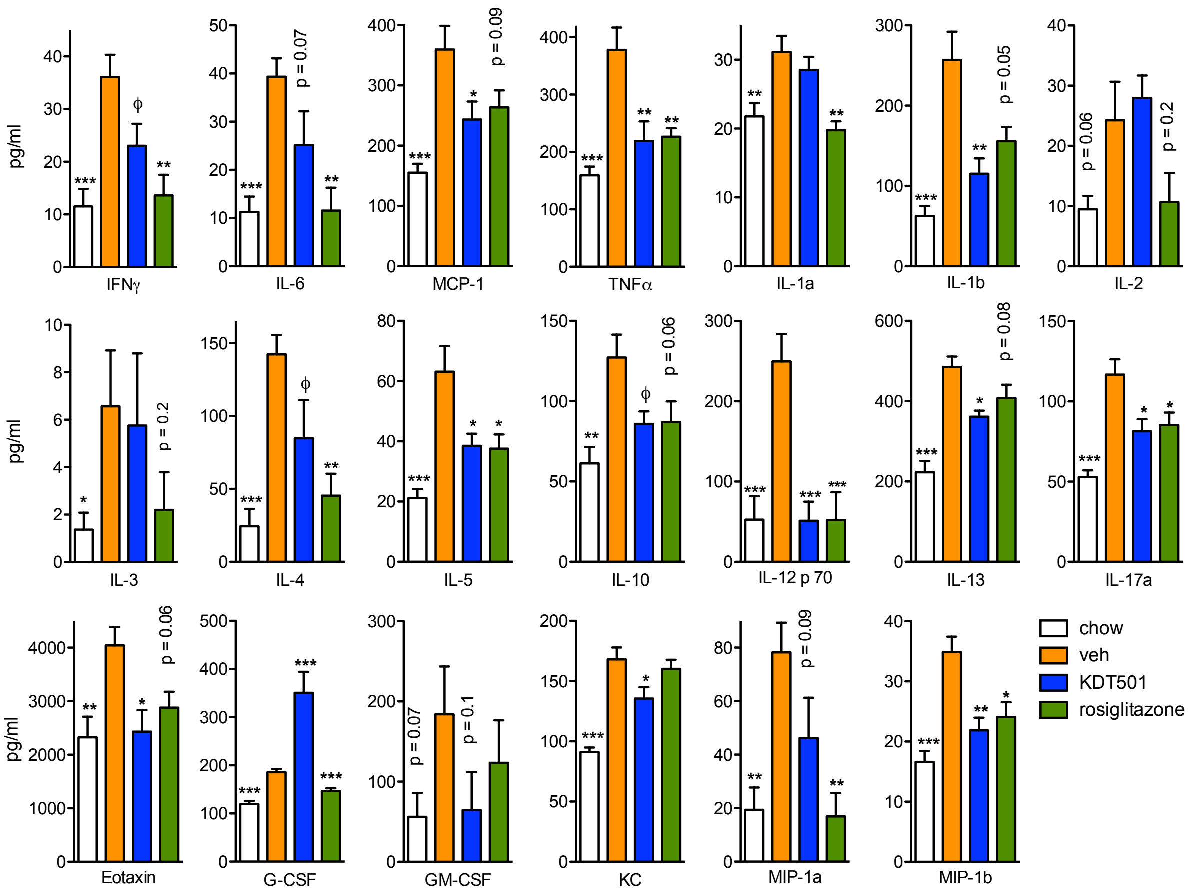

Figure S6. **KDT501 treatment has a broad anti-inflammatory effect**.
Plasma levels of multiple proinflammatory cytokines and chemokines elevated in DIO are reduced by KDT501 treatment. Measurements were performed in vehicle, KDT501, or rosiglitazone-treated DIO mice and matched chow controls (n ≥ 8).
Data are presented as means ± SEM. Statistical significance was calculated using one-way ANOVA (*p < 0.05, **p < 0.01, ***p < 0.001), or Student's t *t*est ($^{\phi}$p < 0.05) relative to vehicle-treated mice.

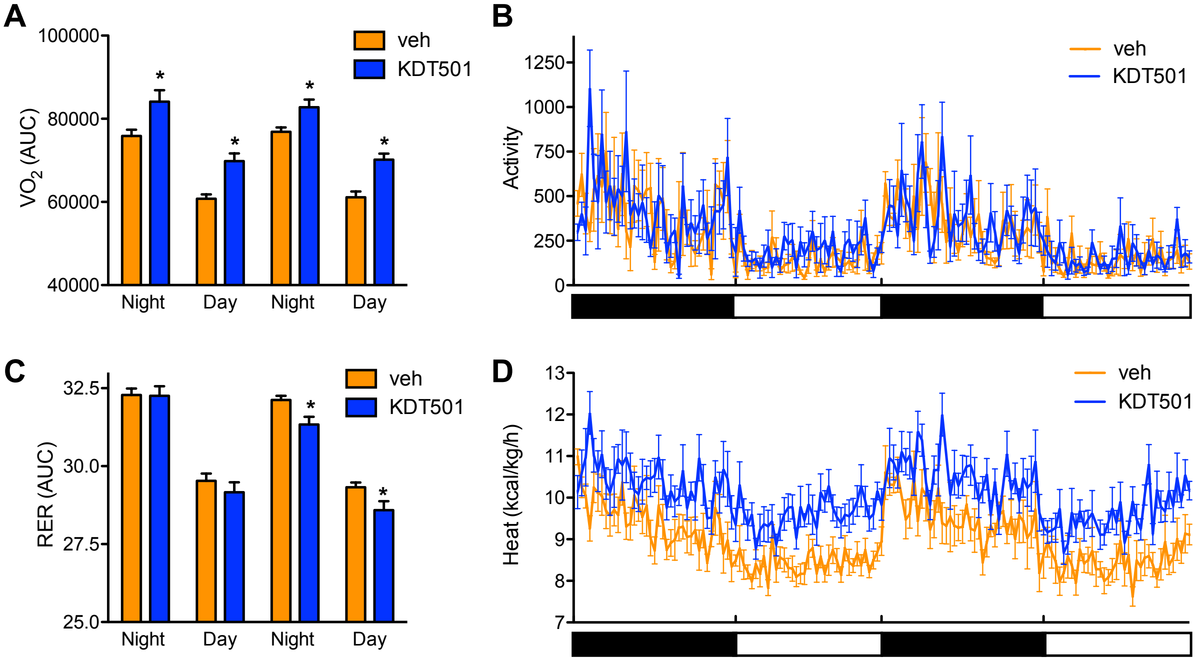

Figure S7. **KDT501 treatment increases energy expenditure and heat production without changes in activity**.

(A to D) Area under curve (AUC) analysis of oxygen consumption rate measurements (A), locomotor activity (B), respiratory exchange ratio (RER) AUC analysis (C), and heat production (D) in mice described in Figure 5 (n = 7).

Data are presented as means ± SEM. Statistical significance was calculated using Student's *t* test. *p < 0.05 relative to vehicle-treated mice.

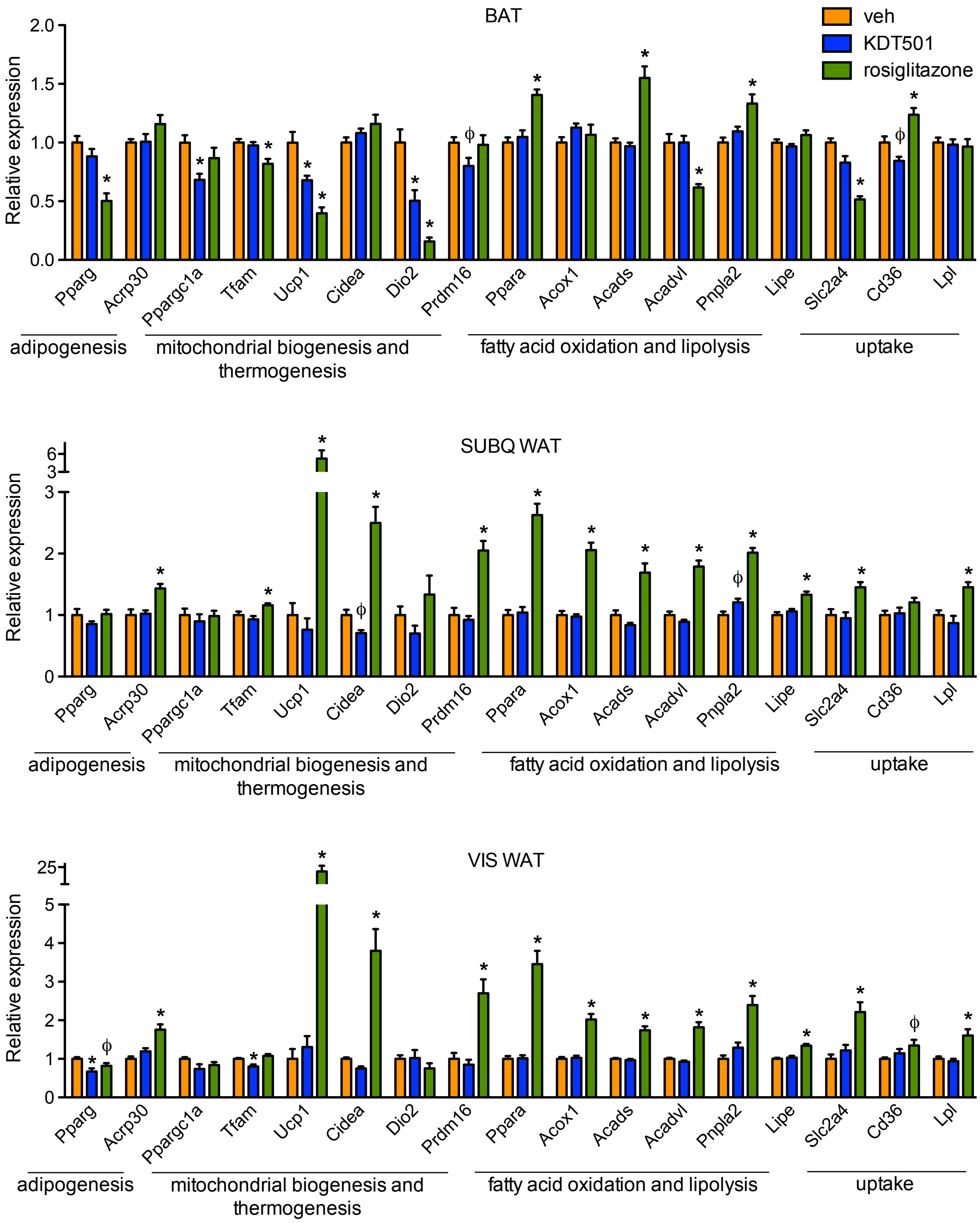

Figure S8. **KDT501 does not alter adipose tissue gene expression**.
RNA levels of genes involved in adipogenesis, mitochondrial biogenesis, thermogenesis, fatty acid oxidation, lipolysis, and substrate uptake were measured in brown adipose tissue (BAT), subcutaneous white adipose tissue (subQ WAT), and visceral WAT (vis WAT) of vehicle, KDT501, and rosiglitazone-treated DIO mice ($n \geq 7$).
Data are presented as means ± SEM. Statistical significance was calculated using one-way ANOVA (*$p < 0.05$) or Student's *t* test ($^{\phi}p < 0.05$) relative to vehicle-treated mice.

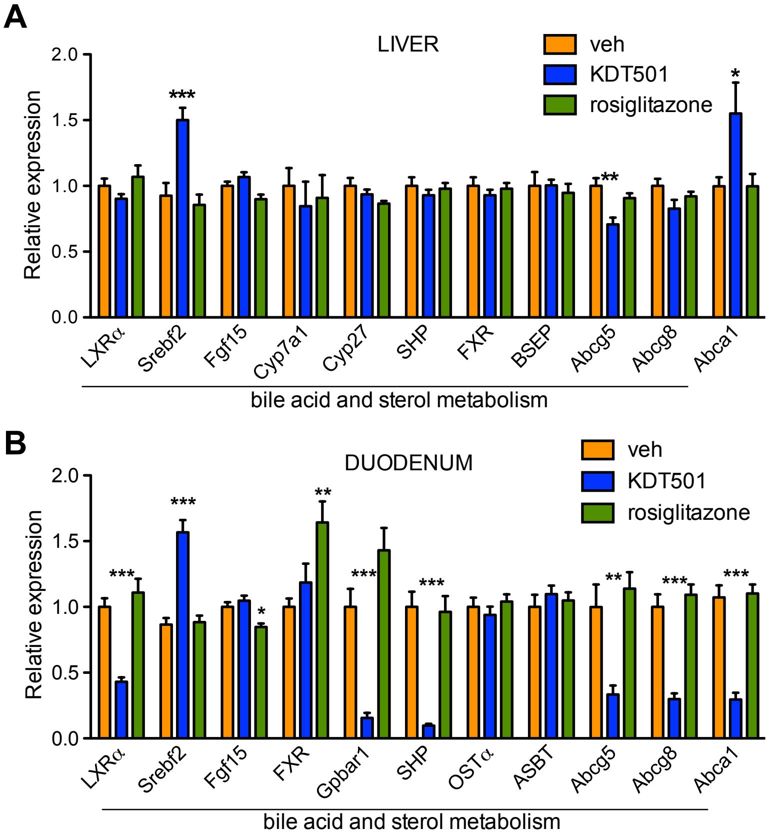

Figure S9. **Effect of KDT501 treatment on expression of bile acid and sterol metabolism genes**.
(A) Hepatic and (B) intestinal RNA levels were measured in vehicle, KDT501, and rosiglitazone-treated DIO mice ($n \geq 7$).
Data are presented as means ± SEM. Statistical significance was calculated using one-way ANOVA. *$p < 0.05$, **$p < 0.01$, ***$p < 0.001$ relative to vehicle-treated mice.

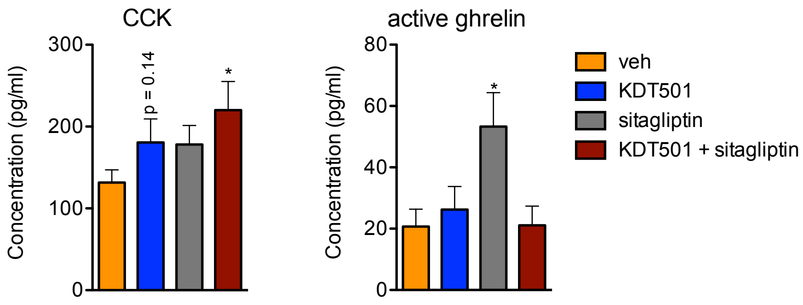

Figure S10. **Effect of short-term KDT501 treatment on plasma ghrelin and CCK levels**. (A) Plasma CCK and (B) active ghrelin levels in DIO mice treated for 4 days with vehicle, 150 mg/kg KDT501, 4 mg/kg sitagliptin, or the combination (n ≥ 6).
Data are presented as means ± SEM. Statistical significance was calculated using one-way ANOVA (*p < 0.05) relative to vehicle-treated mice.

TABLE S1: **PRIMERS AND PROBES FOR QPCR ANALYSIS**.

Gene		Primers or catalog no.
36B4	Forward<br>Reverse<br>Probe	AGATGCAGCAGATCCGCAT<br>GTTCTTGCCCATCAGCACC<br>CGCTCCGAGGGAAGGCCG
Ppara	Forward<br>Reverse<br>Probe	AGTACTTAGGAAGCTGTCCG<br>TCGATGTTCAGGGCACT<br>ATCACAGACACCCTCTCTCCAGC
Acox1	Forward<br>Reverse<br>Probe	CATATGACCCCAAGACCCAAG<br>CATGTAACCCGTAGCACTCC<br>ACTTCCAATCATGCGATAGTCCTGGC
Cpt1a	Forward<br>Reverse<br>Probe	CCAAGTATCTGGCAGTCGA<br>CGCCACAGGACACATAGT<br>CATGGCTCAGACAGTACCTCCTTCA
Acadm	Forward<br>Reverse<br>Probe	ACCCAGATCCTAAAGTACCC<br>CGAAAGCAATTCCTCTGGTG<br>TGGCCCATGTTTAGTTCCTTTTTTCCAA
Acadl	Forward<br>Reverse<br>Probe	GAAACCAGGAACTACGTGAAG<br>GCTGTCCACAAAAGCTCT<br>CACACATACAGACGGTGCAGCATA
Acads	Forward<br>Reverse<br>Probe	ATCCCCAAGGAGAACCTG<br>GCATACTTCACAGCACAATCC<br>CAAAATAGCCATGCAAACCCTGGACA
Acadvl	Forward<br>Reverse<br>Probe	ATCCGAAGCTCAGCCATA<br>CCGTGGCTGCATCTTTAA<br>CCCCCATTACTGATCCAGATCTTGCT
Ppargc1a	Forward<br>Reverse<br>Probe	CATTTGATGCACTGACAGATGGA<br>GTCAGGCATGGAGGAAGGAC<br>CCGTGACCACTGACA ACGAGGCC
Pck1	Forward<br>Reverse<br>Probe	TTGAACTGACAGACTCGCCCT<br>TGCCCATCCGAGTCATGA<br>CCGCATGCTGGCCACCAC
Srebf1	Forward<br>Reverse<br>Probe	ATGGATTGCACATTTGAAGACATGCT<br>CCTGTGTCCCCTGTCTCAC<br>CTTCCCGGGCCTGTTTGACGCCCCCTA
Scd1	Forward<br>Reverse<br>Probe	CTCCTGCTGATGTGCTTCAT<br>AAGGTGCTAACGAACAGGCT<br>TGTTTACAAAAGTCTCGCCCCAGCA
Tfam	Forward<br>Reverse<br>Probe	CACCCAGATGCAAAACTTTCAG<br>CTGCTCTTTATACTTGCTCACAG<br>CCACAGGGCTGCAATTTTCCTAACC
Pnpla2	Forward<br>Reverse<br>Probe	ATATCCCACTTTAGCTCCAAGG<br>CAAGTTGTCTGAAATGCCGC<br>ATTTATCCCGGTGTACTGTGGCCTC
Lipe	Forward<br>Reverse<br>Probe	GCTCCCTTTCCCCGA<br>ATGCAGAGATTCCCACCT<br>CACTGTGACCTGCTTGGTTCAACT
Slc2a4	Forward<br>Reverse<br>Probe	TGTCGCTGGTTTCTCCAACTG<br>CCATACGATCCGCAACATACTG<br>ACCTGTAACTTCATTGTCGGCATGGGTTT

Cd36	Forward<br>Reverse<br>Probe	GCGACATGATTAATGGCACAG<br>GATCCGAACACAGCGTAGATAG<br>CAACAAAAGGTGGAAAGGAGGCTGC
Lpl	Forward<br>Reverse<br>Probe	GCCATGACAAGTCTCTGAAG<br>CTTTCAAACACCCAAACAAGG<br>AGTCTGGCTGACACTGGACAAACA
Pparg	Forward<br>Reverse<br>Probe	TGTTATGGGTGAAACTCTGGG<br>TCTTGTGAAGTGCTCATAGGC<br>CCATGCTCTGGGTCAACAGGAGAAT
Acrp30	Forward<br>Reverse<br>Probe	AGGCATCCCAGGACATC<br>CCTGTCATTCCAACATCTCC<br>CCTTAGGACCAAGAAGACCTGCATCTC
Ucp1	Forward<br>Reverse<br>Probe	GAGCTGGTAACATATGACCTC<br>GAGCTGACAGTAAATGGCA<br>ACAAAATACTGGCAGATGACGTCCC
Cidea	Forward<br>Reverse<br>Probe	CACGCATTTCATGATCTTGG<br>CCTGTATAGGTCGAAGGTGA<br>TTACTACCCGGTGTCCATTTCTGTCC
Dio2	Forward<br>Reverse<br>Probe	CTGTGTCTGGAACAGCTT<br>CACTGGAATTGGGAGCAT<br>CTAGATGCCTACAAACAGGTTAAACTGGGT
Prdm16	Forward<br>Reverse<br>Probe	CAGCACGGTGAAGCCATTC<br>GCGTGCATCCGCTTGTG<br>AGAACTGCGTGTAGGACTTGTGGC
Lxra	Forward<br>Reverse<br>Probe	CAACAGTGTAACAGGCGCT<br>TGCAATGGGCCAAGGC<br>TCAGACCGCCTGCGCGTC
Cyp27	Forward<br>Reverse<br>Probe	GGAGGGCAAGTACCCAATAA<br>GCGATGAAGATCCCATAGGT<br>CCACCGAGACCACAAGGGCC
Abcg5	Forward<br>Reverse<br>Probe	CTCTATCAGCTTGTGGGTG<br>CCACTTATGATACAGGCCATC<br>CTCAGCATGGGAAACAGATTCACAGC
Abcg8	Forward<br>Reverse<br>Probe	TGGTCAGTCCAACACTCT<br>CTCAAACCAAGGCACCT<br>AGAGGCGATGTCCACCTGGTAG
Abca1	Forward<br>Reverse<br>Probe	GGTTTGGAGATGGTTATACAATAGTTGT<br>CCCGGAAACGCAAGTCC<br>CGAATAGCAGGCTCCAACCCTGAC
Hmgcs2	Applied Biosystems	Mm00550050_m1
Hmgcl	Applied Biosystems	Mm00468667_m1
G6pc	Applied Biosystems	Mm00839363_m1
Gck	Applied Biosystems	Mm00439129_m1
Srebf2	Applied Biosystems	Mm01306292_m1
Cyp7a1	Applied Biosystems	Mm00484152_m1
Fgf15	Applied Biosystems	Mm00433278_m1
Gpbar1	Applied Biosystems	Mm04212121_s1
Nr0b2 (SHP)	Applied Biosystems	Mm00442278_m1

Nr1h4 (FXR)	Applied Biosystems	Mm00436425_m1
Abcb11 (BSEP)	Applied Biosystems	Mm00445168_m1
Slc51a (OSTα)	Applied Biosystems	Mm00521530_m1
Slc10a2 (ASBT)	Applied Biosystems	Mm00488258_m1
